# Brain structure and cortical activity changes of new daily persistent headache: multimodal evidence from MEG/sMRI

**DOI:** 10.1186/s10194-023-01581-6

**Published:** 2023-04-26

**Authors:** Dong Qiu, Wei Wang, Yanliang Mei, Hefei Tang, Ziyu Yuan, Peng Zhang, Yaqing Zhang, Xueying Yu, Chunqing Yang, Qun Wang, Yonggang Wang

**Affiliations:** 1 Headache Center, Department of Neurology, Beijing Tiantan Hospital, Capital Medical University, No.119 South Fourth Ring West Road, Fengtai District, 100070 Beijing, China; 2 Department of Neurology, Beijing Tiantan Hospital, Capital Medical University, No.119 South Fourth Ring West Road, Fengtai District, 100070 Beijing, China

**Keywords:** New daily persistent headache, Magnetoencephalography, Structural magnetic resonance imaging, Multimodal

## Abstract

**Background:**

New daily persistent headache (NDPH) is a rare but debilitating primary headache disorder that poses a significant burden on individuals and society. Despite its clinical importance, the underlying pathophysiological mechanisms of NDPH remain unclear. In this study, we aimed to investigate the brain structural changes and neural activity patterns in patients with NDPH using multimodal brain imaging analysis of structural magnetic resonance imaging (sMRI) combined with magnetoencephalography (MEG).

**Methods:**

Twenty-eight patients with NDPH and 37 healthy controls (HCs) were recruited for this study, and their structural and resting-state data were collected by 3.0 Tesla MRI and MEG. We analyzed the brain morphology using voxel-based morphometry and source-based morphometry. In each brain region, MEG sensor signals from 1 to 200 Hz were analyzed using an adapted version of Welch's method. MEG source localization was conducted using the dynamic statistical parametric mapping, and the difference of source distribution between patients with NDPH and HCs was examined.

**Results:**

Our results revealed significant differences in the regional grey matter volume, cortical thickness, and cortical surface area between the two groups. Specifically, compared with HCs, patients with NDPH showed a significant decrease in cortical thickness of the left rostral cortex in the middle frontal gyrus, decreased cortical surface area of the left fusiform gyrus, decreased grey matter volume of the left superior frontal gyrus and the left middle frontal gyrus, and increased grey matter volume of the left calcarine. Furthermore, the power of the whole brain, bilateral frontal lobes, and right temporal lobe in the NDPH group were higher than that in HCs in the ripple frequency band (80-200 Hz). Functional and structural analysis suggested that there were structural changes and abnormal high frequency cortical activity in both frontal and temporal lobes in patients with NDPH.

**Conclusion:**

Our findings indicated that patients with NDPH have abnormalities in brain morphology, such as cortical area, cortical thickness, and grey matter volume, accompanied by abnormal cortical neural activity. Brain structural changes in the frontotemporal cortex and abnormalities in cortical ripple activity may be involved in the pathogenesis of NDPH.

**Supplementary Information:**

The online version contains supplementary material available at 10.1186/s10194-023-01581-6.

## Introduction

New daily persistent headache (NDPH) is a specific type of primary headache, first described as benign persistent daily headache syndrome by Vanast in 1986 [1]. The overall 1-year prevalence of NDPH was 0.03% [2]. The NDPH has unique characteristics, and the diagnostic criteria for *International Classification of Headache Disorders, 3*^*rd*^* Editon* (ICHD-3) clearly state that the patients can accurately recall the onset of the headache and that the headache lasts for more than three months [3]. The headache can persist for many years after treatment, placing a considerable burden on society and individuals.

The headache forms of NDPH could be similar to migraine and tension-type headache, and the types of headaches are heterogeneous. Therefore, some scholars have pointed out whether NDPH can be treated as a separate disease or a companion symptom of other diseases [4]. Consistent precipitating factors (infection, stress, and trauma) were not found in NDPH in the available case reports, and the pathophysiology of NDPH is unclear [5].

Neuroimaging is one of the most outstanding achievements of modern science and is widely used in the study of primary headaches. In 1996, Cicek used MRI analysis and found changes in brain structure in patients with primary headaches [6]. Although this study did not analyze the degree of structural changes in brain regions in detail, it formed the basis for structural imaging related to primary headaches. A study of resting-state functional imaging and structural imaging revealed changes in specific brain regions of patients with NDPH [7]. However, other NDPH structural imaging studies reported no significant brain structural changes in patients with NDPH [8, 9]. Studies on the brain function of NDPH are even more scarce. In adolescents, functional magnetic resonance imaging (fMRI) analysis has shown that patients with NDPH had altered functional connectivity in the amygdala, insula, frontal lobe, and cerebellum [10]. The functional connectivity between brain regions related to pain, emotion, and within cognitive networks were altered, suggesting that NDPH may be associated with brain dysfunction, which provides a direction for future research [7]. However, there were few studies focusing on both the structure and function of patients with NDPH, and the limited evidence only focused on adolescents.

Magnetoencephalography (MEG) is an established method that assesses the magnetic fields generated by electrical activity in neurons [11]. Compared with electroencephalography (EEG), MEG is less affected by soft scalp tissue, skull, and other tissues, so it has a high spatial resolution (about 5 mm). In contrast to fMRI, MEG has a much higher temporal resolution and directly measures electromagnetic signals produced by neurons rather than by changes in blood oxygen that accompany neural activity [12].

Overall, we conducted a multimodal neuroimaging analysis using voxel-based morphometry (VBM) and source-based morphometry (SBM) analysis in structural magnetic resonance imaging (sMRI) combined with MEG. We compared brain structure and function between patients with NDPH and carefully matched healthy controls (HCs). Our hypothesis was that changes in brain morphology in patients with NDPH are accompanied by changes in cortical activity. Additionally, we assessed whether a correlation exists between clinical features and structure in patients with NDPH to shed light on the pathophysiology of NDPH.

## Methods

### Study population

Between October 2020 and October 2022, 30 patients with NDPH and 40 HCs were recruited from the Headache Department, Neurology Centre, Beijing Tiantan Hospital, Capital Medical University. Each patient with NDPH needed to be diagnosed by at least two neurological specialists. Inclusion criteria for patients with NDPH included: (1) Patients who satisfied the definition of NDPH according to ICHD-3 criteria [3]; (2) All participants ranged in age from 14 to 70 years. Exclusion criteria included: (1) Patients combined with other types of primary headache or major systemic diseases; (2) Claustrophobia or metal implants in the body; (3) Poor quality of data (MRI had obvious errors in image segmentation or had a conspicuous abnormality that precluded accurate MEG image registration); (4) Pregnancy or breastfeeding. Patients with NDPH required a headache history registry (headache history, frequency of headache, medication of treatment) and clinical scale assessment at the same time of the MEG acquisition. The Headache Impact Test -6 (HIT-6) score was used to determine the impact intensity of headache [13]. The Patient Health Questionnaire-9 (PHQ-9) and Generalized Anxiety Disorder-7 (GAD-7) scores were used to evaluate anxiety and depression symptoms [14, 15]. The Pittsburgh Sleep Quality Index (PSQI) score was used to assess the quality of sleep [16]. The Visual Analogue Scale (VAS) score was used to assess the level of pain [17].

In addition, age- and sex-matched HCs were recruited. The inclusion criteria for HCs included: (1) No history or family history of headache, no headache episodes in the previous 1 year; (2) Match age and sex to patients of NDPH. Exclusion criteria for HCs include: (1) Major systemic diseases; (2) Claustrophobia or metal implants in the body; (3) Poor quality of data (MRI had obvious errors in image segmentation or had a conspicuous abnormality that precluded accurate MEG image registration); (4) Pregnancy or breastfeeding. The history of severe acute respiratory syndrome coronavirus 2(SARS-CoV2) infection was recorded for all participants.

The study protocol was approved by the Institutional Review Committee of Beijing Tiantan Hospital of Capital Medical University (KY2022-044), which was registered on the https://www.clinicaltrials.gov (unique identifier: NCT05334927). All participants provided informed written consent according to the Declaration of Helsinki.

### MRI data acquisition

All participants were imaged with a 3.0 Tesla MR scanner (GE Healthcare, Milwaukee, WI, USA) by standard head coil at Beijing Tiantan Hospital. This was examined by an experienced neuroradiologist who knew nothing about the participants’ diagnosis. Participants were required to avoid head and neck movements as much as possible, stay awake, relax, and keep eyes closed during the MRI scanning, with earplugs and foam padding to reduce scanner noise and head-movement. Only images without obvious quality problems and pathological changes were included in further analyses. T1-weighted volumetric images were obtained by the 3D BRAVO sequence (coronal acquisition, the field of view (FOV) = 256 mm, acquisition matrix = 256, slice number = 192, flip angle = 15°, TR = 850 ms, TE = 320 ms, voxel size = 1 x 1 x 1.5 mm^3^).

### MEG data acquisition

The Elekta Neuromag 306-channel scanner (Elekta TRIUX ®) was used in this study to record neural activity at 2000 Hz with a low-pass filter set to 660 Hz. Each sensor element consists of a magnetometer and two orthogonal planar gradiometers. Four head position indicator (HPI) coils were placed on the participants’ scalp to indicate head position. Furthermore, coordinates of head points were digitized (Polhemus Fastrak®) for MRI co-registration, including nasion, anterior points in front of the ear points, and about 300 additional points on the scalp. These flags and head position points were used to register the MEG and MRI coordinate systems further. A pair of electrodes were placed and attached to the participants’ chests to capture electrocardiogram synchronized with the MEG recording. Two electrodes were attached above and below eye to detect electro-ocular activity. Five continuous minutes of MEG resting-state data were acquired per participant on the Elekta Neuromag 306-channel scanner. The participant was laid comfortably in the scanner and instructed to remain awake and close eyes, but without performing any specific task. MEG scan would be restarted if the participant falls asleep or has excessive head movements during the scan. Because NDPH was a persistent headache, all patients experienced headaches during the scan.

### MRI processing

#### Voxel-Based Morphometry

The structural image data was converted to NIFTI format by MRIcron software. The SPM12 (https://www.fil.ion.ucl.ac.uk/spm/software/spm12) and CAT12 (https://neuro-jena.github.io/cat/) toolboxes were used for data processing and analysis. All images were first spatially normalized using the standard Montreal Neurological Institute (MNI) template in SPM12. Each normalized image was segmented into grey matter (GM), white matter, and cerebrospinal fluid, and the segmented data was quality checked to exclude participants with obvious errors in image segmentation. The segmented GM images were averaged and smoothed (FWHM = 8 mm), and CAT12 was used to estimate the volume and total intracranial volume (TIV) of different brain tissue classes for statistical analysis.

#### Surface-based morphometry

The FreeSurfer software package was used to analyze the participants’ T1 structural image data (https://surfer.nmr.mgh.harvard.edu), including head movement correction, normalization, removal of non-brain tissue, registration of MNI standard space, segmentation and smoothing of grey-white matter boundaries. The white matter surface was then deformed towards the GM boundary of each vertex. Cortical thickness was calculated based on the distance between the white matter boundary and the GM boundary at each vertex, with visual inspection of the entire cortex of each study participant and manual editing of inaccurate segmentation. According to the Desikan-Killiany Atlas, the brain was divided into 68 cortical regions. Regional average cortical thickness and area estimates were calculated by averaging all vertices.

#### MEG processing

According to the MEG sensor position, 306 channels were divided into eight brain regions (left and right frontal, parietal, occipital, and temporal lobe). All bad channels were detected and excluded before passing the raw data through MaxFilter. A band-pass filter between 1 to 200 Hz was applied. The raw data was removed line noise of 50 Hz power, and downsampled to 1000 Hz. Using independent component analysis (ICA), components such as ocular and cardiac artifacts were removed.

**Fig. 1:**
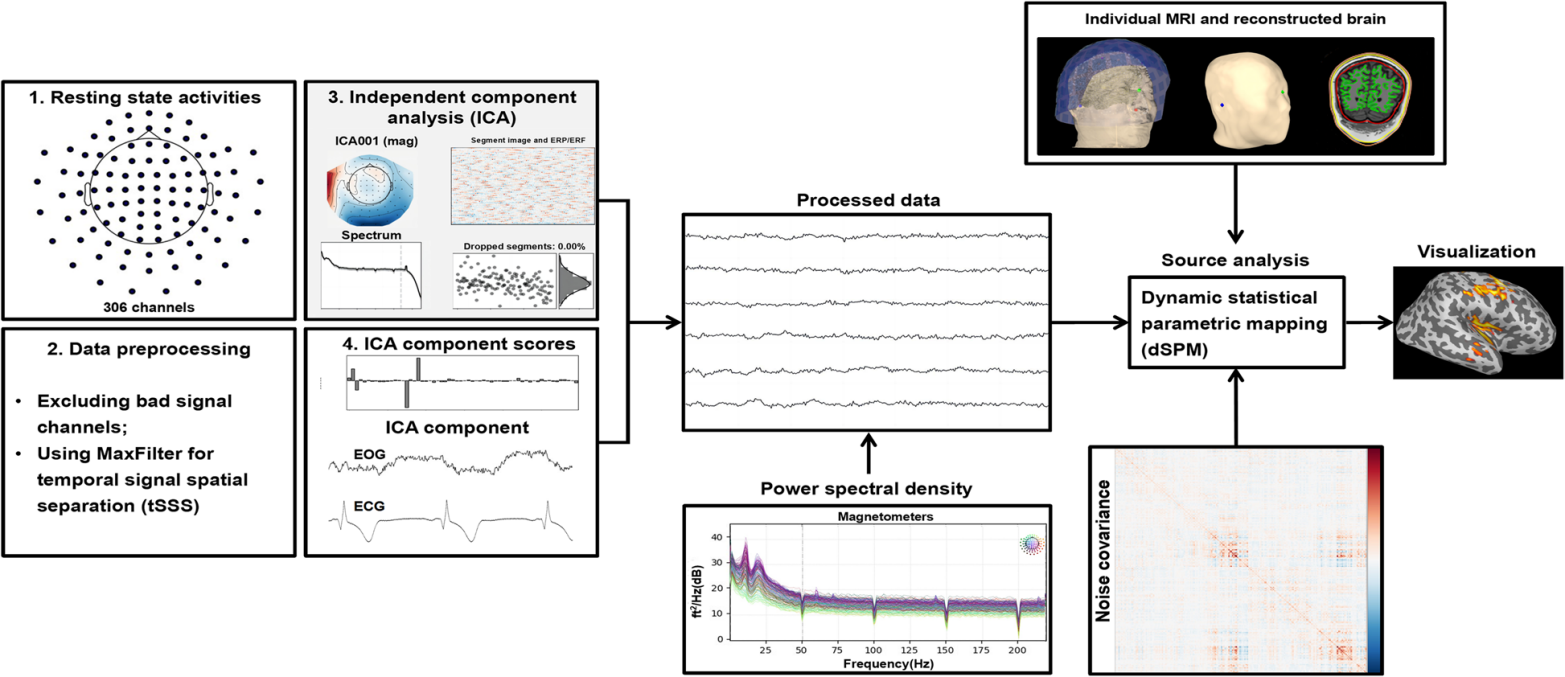
The pipeline of resting-state magnetoencephalographic spectral and source analysis

Welch's algorithm was used to calculate the MEG sensor power spectral density for each participant at the sensor level. In order to quantify brain activation in individual brain regions, the result was calculated using the mean of each frequency band of the sensor. Intergroup comparisons were carried out in six frequency bands: delta (1-4 Hz), theta (4-8 Hz), alpha (8-13 Hz), beta (13-30 Hz), gamma (30-80 Hz), and ripple (80-200 Hz). In the source level, the boundary element model was created and the MRI was co-registered with 3D digitized coordinates. And surface-based source space was computed. The forward solution could be calculated with the magnetic fields and electric potentials at the measurement sensors. Then, the inverse problem and noise covariance were computed. And used dynamic statistical parametric mapping (dSPM) for source estimation. The dSPM method was based on the generalized least-squares or weighted minimum-norm estimate, normalized for noise sensitivity [18, 19]. It transformed the resulting source strengths relative to noises into an F-distributed statistical parameter value at each source. The dSPM F-values were summed for each vertex over the entire time series. The vertex with the largest sum of F-values was identified and defined it as the most active area [20] (Fig. 1).

### Statistical analysis

The sample size was determined based on the available data and previous literature. Assuming no negative correlation between endpoints, a sample size of 65 cases (37 HC group and 28 NDPH group) would provide 85% power to reject the null hypothesis equal means at a two-sided alpha of 0.05. IBM SPSS 22.0 was used to perform the statistical analysis. Mean ± standard deviation and median with interquartile range were described as the normally and non-normally distributed data, respectively. Categorical variables were expressed as number (percentage). The comparison of continuous and categorical variables between the patient and HC groups was carried out using independent sample t-test and chi-square test respectively in demographic and clinical data. Before the independent sample t-test, we test the normality of the data by the Kruskal–Wallis test. The Mann–Whitney U test was applied to compare non-normally distributed data between the groups. Correlations between GM, cortical thickness, surface area, and demographic data were calculated using Pearson’s correlation (statistical tests were carried out at p < 0.002 [0.05/25] significance level after Bonferroni correction). All statistical tests are two-tailed tests, with P < 0.05 indicating statistical significance. Covariates consist of age, sex, and TIV, and independent sample t-tests were used to compare GM volume between groups. Using the general linear model (GLM), age and sex were included as covariates, detected average cortical thickness and the surface area of 68 cerebral cortical regions between groups. This study used an uncorrected threshold of P < 0.01 for initial vertex-wise comparisons. To correct for multiple comparisons, a Monte Carlo simulation with 10,000 times was performed. Only the cluster with a continuous extent of 100 vertices and a significance threshold of P < 0.05 in the cluster level were reported. The independent sample t-test was used to compare the power spectral density of the MEG sensor between groups, and FDR correction was performed. The difference in the distribution of magnetic source activity between patients and the HC groups was compared by Fisher's exact test (α = 0.05).

## Results

### Clinical characteristics and demographics

Thirty patients (14 males and 16 females) with NDPH were recruited and willing to participate in this study. A total of 40 well-matched HCs were recruited. Two patients and 3 HCs were excluded due to poor quality of data, segmentation errors. Finally, twenty-eight patients with NDPH (14 males, 14 females) and 37 HCs (16 males, 21 females) were included in this study. There were no significant differences in age and sex between the groups. All participants were right-handed and no SARS-CoV2 infection before developing headache. The median onset time of NDPH was 17 years, and the median duration was 3.5 years, ranging from 1 to 14 years. The clinical manifestations of the NDPH group were mainly nausea and vomiting (7.14%), photophobia (39.28%), and phonophobia (53.57%). Three patients were unwilling to cooperate with the completion of HIT-6, GAD-7, and PSQI scale assessments, and two patients with NDPH were reluctant to cooperate with the completion of the PHQ-9 scale evaluation. Demographic and clinical features are summarized in Table 1.

**Table 1:** Participants’ demographics and clinical characteristics

	**Healthy Controls (*n* = 37)**	**NDPH (*n* = 28)**	***p*-value**
Age, years	33.00 (25.50, 43.50)	33.00 (17.00, 59.00)	0.926
Sex (male/female)	16/21 (43.24/56.76%)	14/14 (50.00/50.00%)	0.588
BMI (kg/m^2^)	22.45 ± 3.04	24.45 ± 3.88	0.023
Headache laterality, n (%)			
Unilateral	NA	10 (35.71%)	NA
Bilateral	NA	18 (64.29%)	NA
Location of headache, n (%)			
Frontal region	NA	9 (32.14%)	NA
Temporal region	NA	15 (53.57%)	NA
Parietal region	NA	10 (35.71%)	NA
Occipital region	NA	5 (17.86%)	NA
Periorbital region	NA	1 (3.57%)	NA
Nausea, vomiting, n (%)	NA	2 (7.14%)	NA
Photophobia, n (%)	NA	11 (39.28%)	NA
Phonophobia, n (%)	NA	15 (53.57%)	NA
Age at onset, years	NA	17.00 (13.00, 39.55)	NA
Disease duration, years	NA	3.50 (1.00, 14.00)	NA
VAS score (0–10)	NA	4.00 (3.00, 7.00)	NA
HIT-6 score (36–78) ^a^	NA	62.64 ± 10.94	NA
PHQ-9 score (0–27) ^b^	NA	10.04 ± 6.89	NA
GAD-7 score (0–21) ^a^	NA	6.80 ± 5.26	NA
PSQI score (0–21) ^a^	NA	10.20 ± 5.03	NA

Data are presented as mean ± standard deviation or as median [interquartile range, IQR]

*NDPH* New daily persistent headache; *NA* Not applicable; *BMI* Body mass index; *VAS* Visual analogue scale; *HIT-6* Headache Impact Test-6; *PHQ-9* Patient Health Questionnaire-9; *GAD-7* Generalized Anxiety Disorder-7; *PSQI* Pittsburgh Sleep Quality Index

^a^Scales data were obtained from twenty-five patients with NDPH

^b^Scales data were obtained from twenty-six patients with NDPH

### Brain morphological changes in patients with NDPH

**Fig. 2:**
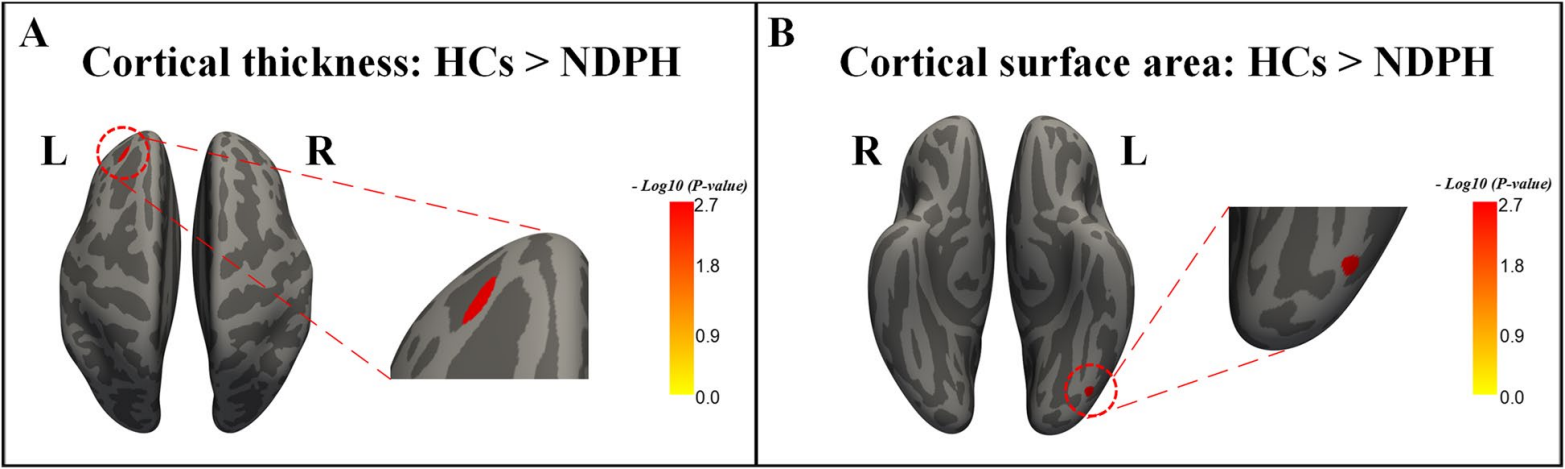
The abnormal cortical thickness and surface area in NDPH. **A** The NDPH group showed significantly decreased cortical thickness in the left rostral middle frontal gyrus compared with HCs. **B** The NDPH group showed significantly decreased cortical surface area in the left fusiform gyrus compared with HCs. HCs, healthy controls; NDPH, new daily persistent headache;  L Left; R Right

**Fig. 3:**
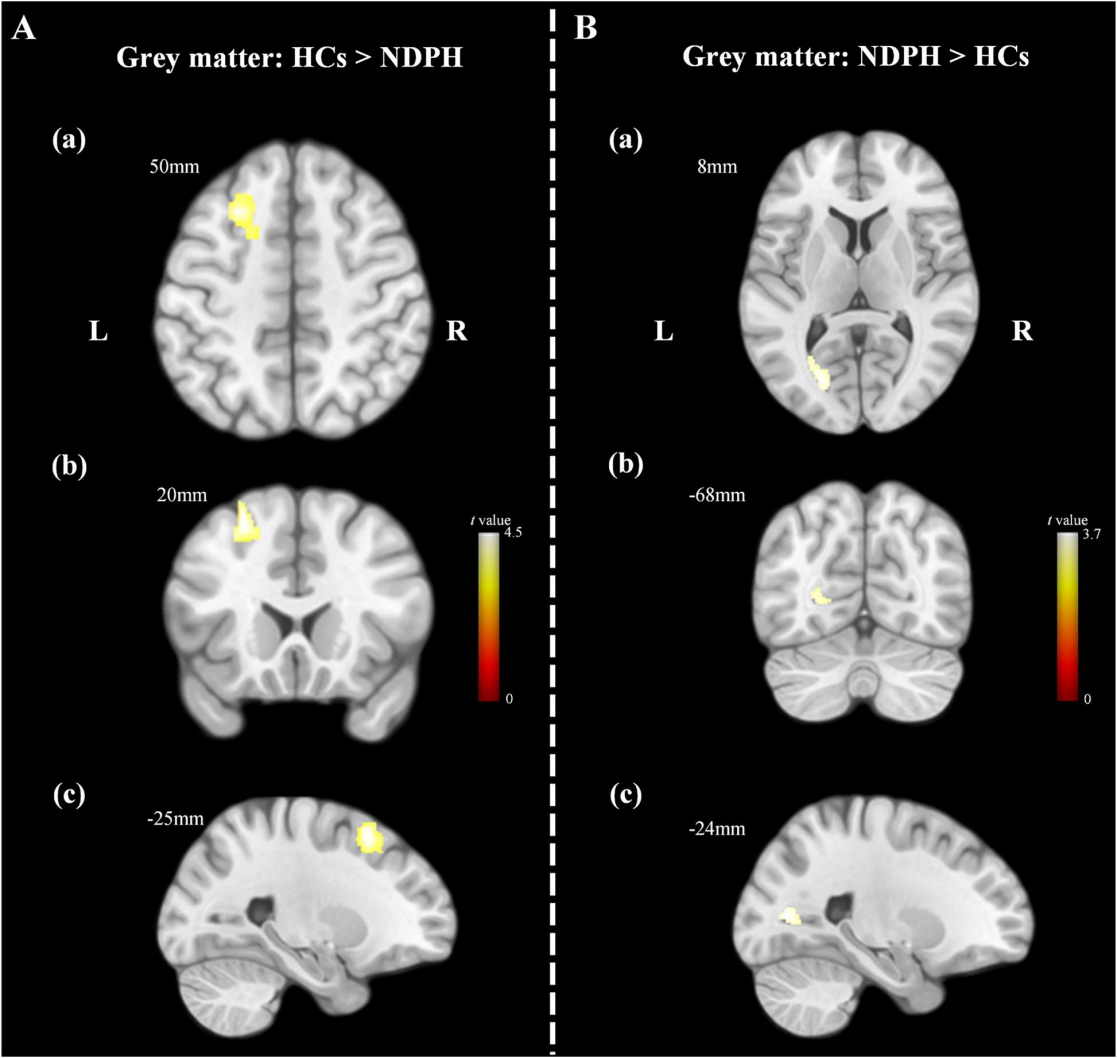
The abnormal grey matter volume in NDPH.** A** The NDPH group showed decreased grey matter volume in the left superior frontal and middle frontal gyrus compared with HCs. **B** The NDPH group showed increased grey matter volume in the calcarine compared with HCs. HCs, healthy controls; NDPH, new daily persistent headache;  L Left; R Right

Compared with HCs, patients with NDPH had decreased cortical thickness of the left rostral middle frontal gyrus (left, MNI x/y/z = -24.6/35.5/28.8, cluster size = 116.78, *p* = 0.002), decreased cortical surface area of the left fusiform gyrus (left, MNI x/y/z = -40.7/-72.1/-13.9, cluster size = 48.36, *p* = 0.006) (Table 2) (Fig. 2), decreased GM volume of the left superior frontal gyrus and the left middle frontal gyrus (left, MNI x/y/z = -21.0/21.0/56.0, cluster size = 515, t = -3.23, *p* = 0.002), increased GM volume of the left calcarine (left, MNI x/ y/z = -24.0/-74.0/9.0, cluster size = 308, t = 3.69, *p* = 0.032) (Table 3) (Fig. 3).

**Table 2:** Brain region with cortical thickness and surface area changes in patients with NDPH

Brain region	Side	MNI coordinates			Cluster size (mm^2^)	CWP
		x	y	z		
Cortical thickness; NDPH < HCs						
Rostral middle frontal gyrus	L	-24.6	35.5	28.8	116.78	0.002**
Cortical surface area; NDPH < HCs						
Fusiform gyrus	L	-40.7	-72.1	-13.9	48.36	0.006**

Brain region localizations were performed using Desikan-Killiany atlas

*MNI* Montreal Neurological Institute; *HCs* Healthy controls; *NDPH* New daily persistent headache; *L* Left; *CWP* Cluster-wise corrected *p*-value

**, *p* < 0.01

**Table 3:** Brain region with grey matter volume changes in patients with NDPH

Brain region	Side	MNI coordinates			Peak *t*-valve	Cluster size (voxels)	Cluster level *P*_FWE corr_
		x	y	z			
Grey matter volume; NDPH < HCs							
Cluster 1		-21.0	21.0	56.0	-3.23	515	0.002**
Superior frontal gyrus	L						
Middle frontal gyrus	L						
Grey matter volume; NDPH > HCs							
Cluster 1		-24.0	-74.0	9.0	3.69	308	0.032*
Calcarine	L						

Brain region localizations were performed using automatic anatomical labeling atlas, and the number of voxels of the anatomical regions in which the cluster extends to were reported

*MNI* Montreal Neurological Institute; *HCs* Healthy controls; *NDPH* New daily persistent headache; *L* Left; *FWE corr.* Family wise error correction

^*^, *p* < 0.05; **, *p* < 0.01

### MEG analysis

**Fig. 4:**
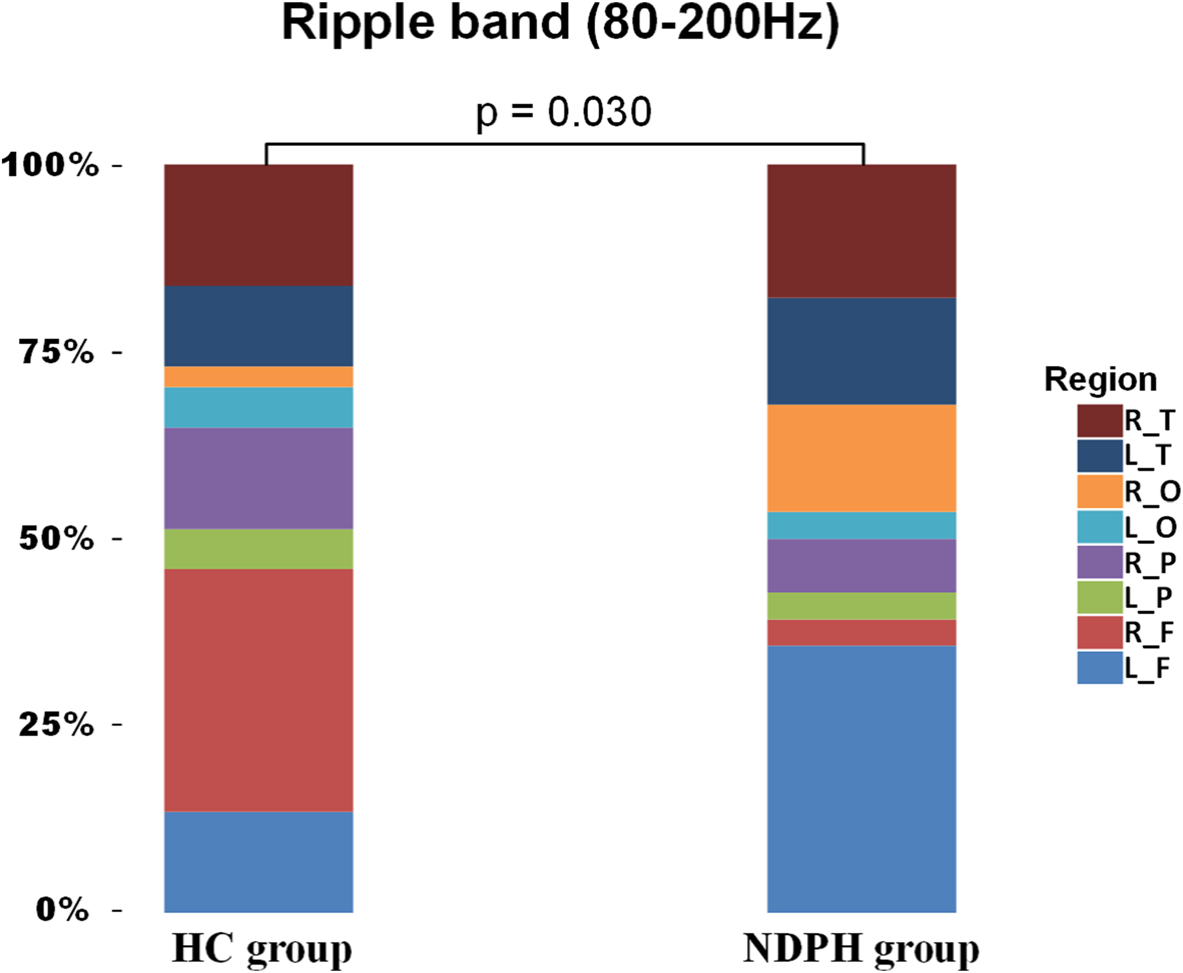
The distribution of neuromagnetic activity in the ripple band. The distribution of the neuromagnetic activity was significantly different between the HC and NDPH groups. HC, healthy control; NDPH, new daily persistent headache. L_F, left frontal lobe; R_F, right frontal lobe; L_P, left parietal lobe; R_P, right parietal lobe; L_O, left occipital lobe; R_O, right occipital lobe; L_T, left temporal lobe; R_T, right temporal lobe

There was no significant difference in delta and beta bands power between patients with NDPH and HCs. Patients' resting-state neural oscillations altered, with an increase in theta band power in the left frontal (*p* = 0.041) and right frontal (*p* = 0.021). Patients with NDPH showed increased alpha band power in the left frontal (*p* = 0.021), increased gamma band power in the right frontal lobe (*p* = 0.010), and right temporal lobe (*p* = 0.006). The NDPH group had significantly increased ripple band power of the whole brain (*p* = 0.006), left frontal lobe (*p* = 0.044), right frontal lobe (*p* < 0.001), and right temporal lobe (*p* = 0.002) (Table 4). The statistical results of the power spectrum between all groups are shown in Table S1. In the source analysis, there was a significant difference in the distribution of neuromagnetic activity regions between the NDPH group and the HC group (*p* = 0.030) in the ripple band (Fig. 4). In other frequency bands, the distribution of neuromagnetic activity regions was not statistically significant (Table S2).

**Table 4:** The power spectral density of each frequency band

Frequency	Region	Power (dB) of HCs	Power (dB) of NDPH	*p*-value
Theta (4-8 Hz)	L_F	15.23 ± 2.31	16.56 ± 2.23	0.041*
	R_F	15.63 ± 2.52	17.19 ± 2.23	0.021*
Alpha (8-13 Hz)	L_F	13.58 ± 2.37	15.01 ± 2.00	0.021*
Gamma (30-80 Hz)	R_F	5.47 ± 1.26	6.38 ± 1.17	0.010*
	R_T	6.20 ± 1.19	7.10 ± 0.80	0.006**
Ripple (80-200 Hz)	All	3.85 ± 0.68	4.31 ± 0.48	0.006**
	L_F	3.51 ± 1.52	4.23 ± 1.06	0.044*
	R_F	3.17 ± 1.19	4.30 ± 1.07	< 0.001***
	R_T	3.87 ± 1.11	4.77 ± 0.69	0.002**

*HCs* Healthy controls; *NDPH* New daily persistent headache; *All* All brain regions; *L_F* Left frontal lobe; *R_F* Right frontal lobe; *R_T* Right temporal lobe

^*^, *p* < 0.05; **, *p* < 0.01; ***,* p* < 0.001

### Correlation analysis

The relationship between brain regions with statistically different structures and clinical features in all patients with NDPH was as follows. The GM volume of the left middle frontal gyrus was negatively correlated with the HIT-6 score (*p* = 0.030, *r* = -0.443, *n* = 25) and PSQI score (*p* = 0.021, *r* = -0.467, *n* = 25). The GM volume of the left superior frontal gyrus was negatively correlated with the HIT-6 score (*p* = 0.022, *r* = -0.465, *n* = 25) and PSQI score (*p* = 0.034, *r* = -0.433, *n* = 25). The cortical thickness of the left rostral middle frontal gyrus was negatively correlated with the VAS score (*p* = 0.049, *r* = -0.368, *n* = 28) and PSQI score (*p* = 0.039, *r* = -0.415, *n* = 25). The cortical surface area of left fusiform gyrus was negatively correlated with the PHQ-9 score (*p* = 0.043, *r* = -0.400, *n* = 26). Clinical features, including VAS, PHQ-9, GAD-7, HIT-6, and PSQI scores, were not significantly correlated with all brain morphology data after Bonferroni correction.

## Discussion

In this study, we performed the first multimodal imaging analysis of structural magnetic resonance combined with MEG in NDPH, a rare headache disease. We found that patients with NDPH had abnormalities of brain structure and cortical activity. The main findings of this study included: (1) Compared to HCs, the NDPH group had significantly decreased cortical thickness of the left rostral middle frontal gyrus, the decreased cortical surface area of the left fusiform gyrus, decreased GM volume of the left middle and superior frontal gyrus, and increased GM volume of left calcarine; (2) As expected, our study showed significant difference in theta, alpha, gamma and ripple bands power between patients with NDPH and HCs. Notably, our study found that the median time to onset of NDPH was 17 years and the minimum age of onset was 11 years. This age of onset was smaller than that reported in previous literatures, but considering that the population in this study was mainly Asian and the case number of previous studies was small, the results of this study were a good supplement to the epidemiology of NDPH [21, 22]. Our Results provide insights into the brain mechanisms that might be related to NDPH and open new avenues for biomarker and personalized treatment research.

### Brain morphological signature of NDPH

In this study, VBM/SBM analysis showed multiple brain morphological changes in patients with NDPH. In contrast to a previous comprehensive VBM/SBM analysis study on patients with NDPH [8], we reported decreased cortical thickness and GM volume in the left frontal lobe, and similar results were reported in a previous NDPH sMRI study [10]. The frontal lobe involves pain regulation, cognitive control, and executive function [23, 24]. Decreased cortical thickness of the middle frontal in patients with NDPH implies increased pain sensitivity and leads to persistent pain. In a function neuroimaging study on chronic inflammatory pain, patients have a significantly negative correlation between the ventrolateral prefrontal cortex activity and pain sensitivity [25]. NDPH can be characterized by migraine-like headaches, and sometimes it is difficult to distinguish between the two disorders. Elevated levels of the pro-inflammatory cytokines IL-6, IL-8, and TNF-α are commonly observed in patients with migraine [26]. Although the etiology of NDPH is not fully understood, previous clinical evidence suggested that infection and inflammation may be important predisposing factors for NDPH [27]. Interestingly, decreased cortical thickness of the middle frontal gyrus was associated with increased pain intensity and poorer quality of sleep [28]. The middle frontal gyrus is involved in forming slow waves, the major electrophysiological features of non-rapid eye movement [29]. During slow-wave sleep, dopamine metabolism in the striatum and the thalamus are reduced, reducing pain sensitivity [30]. This would explain the correlation between cortical thickness of the middle frontal gyrus and sleep quality [31]. Decreased GM volume of the middle frontal gyrus and superior frontal gyrus, especially on the left side, is common in adults and children with repeated and prolonged exposure to pain. In a VBM study of migraine, there was also a decrease of GM volume in the superfrontal and middle frontal gyrus in the migraine group [32]. The superior frontal and middle frontal gyrus are involved in integrating and processing pain signals. Taken in isolation, they are also related to executive function and correlated with the patient's quality of life [33].

In addition, we identified the decreased cortical surface area of the fusiform gyrus in patients, which was associated with body and facial recognition [34]. Bonanno et al. recently confirmed that patients with migraine without aura had decreased GM volume in bilateral fusiform gyrus [35]. A fMRI study also reported that abnormal fusiform gyrus activity was negatively correlated to the subject's pain intensity [36, 37]. In clinical scale analysis, a decrease in the surface area of the fusiform gyrus correlates with the degree of depression. We suspected hyperconnectivity between the fusiform gyrus, the left amygdalostriatal transition area, and the basolateral amygdala. Structural changes in the fusiform gyrus may cause amygdala subregional network dysfunction, leading to different subtypes of mood disorders [38].

We found that 39.28% of NDPH group had photophobia symptoms and their GM volume of calcarine increased. The brain perfusion of occipital in patients with NDPH was altered in our previous study, and we suggested that patients with NDPH may have abnormal activation of the visual system [39, 40]. This is not surprising given the visual pathway's established role in nociceptive pathways. Changes in the visual cortex had also been observed in other headache disorders. There was increased cortex thickness of visual cortex in patients with migraine with aura and might be involved in increased density of neurons [41, 42].

### The resting state cortical oscillation of patients with NDPH

Neural oscillation is crucial for understanding pain. It plays an important role in integrating and separating activity in different brain regions, which is essential for brain function, including pain. Since Dr. Berger used a galvanometer to observe changes in voltage in human skulls, brain activity in different frequency bands have been gradually recognized [43, 44]. Studies of neural oscillations associated with pain have identified several key bands, particularly the theta, beta and gamma bands were associated with nociceptive processes.

Our data supported that the theta band power of the bilateral frontal lobe was significantly increased in patients with NDPH. Theta oscillations originating in the hippocampus are associated with work memory, episodic memory, and pain. Abnormal theta oscillation in the sensorimotor cortex has been shown to cause increased pain sensitivity in animal experiments [45–47]. In patients with fibromyalgia, abnormal theta band power may be associated with dysfunction of sensory processing in chronic pain [48].

We observed increased alpha band power in the left frontal lobe in patients. Abnormal alpha band power is also shown in many chronic pain diseases, and it has also been associated with pain experience [49]. Recent evidence had suggested that alpha band power and peak alpha frequency are associated with pain perception [50]. The MEG/EEG analysis of patients with neuropathic pain found that the neural oscillations were generally abnormal, and the abnormal brain regions were mainly concentrated in the ascending nociceptive pathway, default mode network and salience network [51, 52].

We observed increased gamma band power in the right frontal lobe and right temporal lobe in patients with NDPH. Our results were similar to a previous study which found a significant increase in gamma oscillatory power in frontal and temporal regions in patients with migraine [53]. Gamma oscillations are generated in neuronal networks involving excitatory pyramidal cells and inhibitory gamma aminobutyric acid (GABA)-energic interneurons. Zhou et al. found that increased activity of gamma oscillation in the prefrontal cortex and cerebellar may be a characteristic marker in patients with chronic neuropathic pain [54]. Because the increased gamma band power was associated with an increased pain sensitivity, our findings may provide evidence of pain chronicity in patients with NDPH [55].

Finally, our study showed that the ripple band power of the whole brain, bilateral frontal, and right temporal lobe of patients with NDPH was significantly increased. Physiological ripple oscillations are involved in the memory consolidation process. Ripple oscillations have also been observed in patients with epilepsy and brain tumors [56]. The ripple oscillations in the hippocampus can be consistent with previous experiences. Interestingly, we also observed that patients with NDPH could still clearly recall their first onset years later [57]. Compared with HCs, we found that the distribution of cortical neural activity of patients with NDPH altered in the ripple band by the source-level analysis. This change suggested the presence of whole-brain dysfunction in patients of NDPH.

### Study limitations and future directions

This is the first study that combined sMRI and MEG to explore characteristics of brain structure, resting-state neural oscillation in patients with NDPH. Here we note some limitations in this study. The sample size was relatively small, and our preliminary results need to be verified with more data. Another limitation of this study was the lack of clinical scale information of HC group and a few patients of NDPH. We only analyzed resting-state MEG data, future studies could focus on changes in brain function in patients with NDPH while conducting cognitive tasks to further assess abnormalities in pain regulation circuits.

## Conclusion

Our study expands the research on the brain structure and function related to NDPH. This study showed that patients with NDPH have changes in brain morphology such as cortical surface area, cortical thickness, and GM volume, accompanied by abnormal cortical neural activity. Brain structural changes in the frontotemporal cortex and abnormalities in cortical ripple activity may be involved in the pathogenesis of NDPH. These results provide new insights into the mechanisms of NDPH and may aid in the development of new treatment strategies.

## Supplementary Information

**Additional file 1: Table S1.** The power spectral density of each frequency band. HCs, healthy controls; NDPH, new daily persistent headache; All, all brain regions; L_F, left frontal lobe; R_F, right frontal lobe; L_P, left parietal lobe; R_P, right parietal lobe; L_O, left occipital lobe; R_O, right occipital lobe; L_T, left temporal lobe; R_T, right temporal lobe.

**Additional file 2: Table S2.** The number of NDPH and HCs in different neuromagnetic activity areas. HCs, healthy controls; NDPH, new daily persistent headache; All, all brain regions; L_F, left frontal lobe; R_F, right frontal lobe; L_P, left parietal lobe; R_P, right parietal lobe; L_O, left occipital lobe; R_O, right occipital lobe; L_T, left temporal lobe; R_T, right temporal lobe; *, p<0.05.

## Data Availability

The data that support the findings of this study are available from the corresponding author, upon reasonable request.

## Abbreviations

dSPM: Dynamic statistical parametric mapping
FWE: Family wise error
fMRI: Functional magnetic resonance imaging
GAD-7: Generalized Anxiety Disorder-7
GLM: General linear model
GM: Grey matter
GABA: Gamma aminobutyric acid
HCs: Healthy controls
HIT-6: Headache Impact Test-6
HPI: Headache position indicator
ICA: Independent component analysis
MEG: Magnetoencephalography
MNI: Montreal Neurological Institute
PHQ-9: Patient Health Questionnaire-9
PSQI: Pittsburgh Sleep Quality Index
SARS-CoV2: Severe acute respiratory syndrome coronavirus 2
SBM: Source-based morphometry
sMRI: Structural magnetic resonance imaging
TIV: Total intracranial volume
VBM: Voxel-based morphometry
VAS: Visual Analogue Scale
