# Supplementary material for: Brain structure and cortical activity changes of new daily persistent headache: multimodal evidence from MEG/sMRI
Source: J Headache Pain. 2023 Apr 26;24(1):45. doi: 10.1186/s10194-023-01581-6 (PMC10129440; doi:10.1186/s10194-023-01581-6)
Supplement: Supplementary file 1 — Additional file 1: Table S1. The power spectral density of each frequency band. HCs, healthy controls; NDPH, new daily persistent headache; All, all brain regions; L_F, left frontal lobe; R_F, right frontal lobe; L_P, left parietal lobe; R_P, right parietal lobe; L_O, left occipital lobe; R_O, right occipital lobe; L_T, left temporal lobe; R_T, right temporal lobe. [file 10194_2023_1581_MOESM1_ESM.docx]

| **Table S1** **The power spectral density of each frequency band** | | | | |
| --- | --- | --- | --- | --- |
| **Frequency** | **Region** | **Power (dB) of HCs** | **Power (dB) of NDPH** | ***p*-value** |
| Delta (1-4Hz) | ALL | 20.52±1.81 | 21.04±2.00 | 0.341 |
|  | L_F | 19.41±2.57 | 20.06±1.98 | 0.299 |
|  | R_F | 20.12±3.00 | 20.98±2.39 | 0.249 |
|  | L_P | 21.20±1.84 | 21.39±2.61 | 0.784 |
|  | R_P | 21.10±2.36 | 21.24±2.96 | 0.853 |
|  | L_O | 19.88±2.02 | 20.25±2.92 | 0.624 |
|  | R_O | 19.25±2.10 | 20.14±2.64 | 0.207 |
|  | L_T | 22.02±2.30 | 21.91±2.25 | 0.872 |
|  | R_T | 21.17±2.47 | 22.37±2.36 | 0.082 |
| Theta (4-8Hz) | All | 16.64±1.94 | 17.83±2.35 | 0.061 |
|  | L_P | 17.72±2.18 | 18.29±2.85 | 0.441 |
|  | R_P | 17.92±2.03 | 18.73±3.04 | 0.296 |
|  | L_O | 16.13±2.54 | 17.11±3.11 | 0.238 |
|  | R_O | 15.54±2.38 | 16.94±3.29 | 0.100 |
|  | L_T | 17.43±2.50 | 18.80±2.77 | 0.075 |
|  | R_T | 17.49±2.32 | 18.99±2.97 | 0.059 |
| Alpha (8-13Hz) | All | 16.47±2.53 | 17.24±2.52 | 0.28 |
|  | R_F | 14.05±2.62 | 15.28±2.34 | 0.079 |
|  | L_P | 17.99±2.75 | 17.69±3.01 | 0.713 |
|  | R_P | 17.85±3.28 | 17.99±3.42 | 0.882 |
|  | L_O | 16.60±3.48 | 17.49±3.58 | 0.377 |
|  | R_O | 16.74±3.34 | 17.62±3.98 | 0.408 |
|  | L_T | 17.46±2.91 | 18.09±2.72 | 0.429 |
|  | R_T | 17.50±2.69 | 18.79±2.99 | 0.119 |
| Beta (13-30Hz) | All | 12.53±2.18 | 12.64±1.84 | 0.85 |
|  | L_F | 10.97±2.19 | 11.28±2.07 | 0.599 |
|  | R_F | 11.09±2.37 | 11.77±2.18 | 0.291 |
|  | L_P | 14.50±3.27 | 13.59±2.76 | 0.28 |
|  | R_P | 14.36±3.18 | 13.88±2.66 | 0.556 |
|  | L_O | 11.77±2.40 | 11.71±2.18 | 0.933 |
|  | R_O | 11.45±2.29 | 11.90±2.31 | 0.49 |
|  | L_T | 13.20±2.42 | 13.10±2.11 | 0.877 |
|  | R_T | 12.93±2.24 | 13.87±2.07 | 0.125 |
| Gamma (30-80Hz) | All | 6.17±0.68 | 6.50±0.58 | 0.064 |
|  | L_F | 5.67±1.33 | 6.25±1.08 | 0.087 |
|  | L_P | 7.31±0.94 | 7.09±1.17 | 0.461 |
|  | R_P | 6.98±0.98 | 7.05±1.14 | 0.834 |
|  | L_O | 5.62±1.06 | 5.71±0.97 | 0.757 |
|  | R_O | 5.25±1.07 | 5.66±0.96 | 0.151 |
|  | L_T | 6.85±1.48 | 6.77±0.79 | 0.827 |
| Ripple (80-200Hz) | L_P | 4.50±0.68 | 4.58±0.94 | 0.74 |
|  | R_P | 4.12±0.59 | 4.47±0.76 | 0.088 |
|  | L_O | 3.71±0.96 | 3.80±0.83 | 0.731 |
|  | R_O | 3.39±0.91 | 3.67±0.78 | 0.235 |
|  | L_T | 4.53±1.81 | 4.62±0.83 | 0.84 |
| HCs, healthy controls; NDPH, new daily persistent headache; All, all brain regions; L_F, left frontal lobe; R_F, right frontal lobe; L_P, left parietal lobe; R_P, right parietal lobe; L_O, left occipital lobe; R_O, right occipital lobe; L_T, left temporal lobe; R_T, right temporal lobe. | | | | |
