# Supplementary material for: Brain structure and cortical activity changes of new daily persistent headache: multimodal evidence from MEG/sMRI
Source: J Headache Pain. 2023 Apr 26;24(1):45. doi: 10.1186/s10194-023-01581-6 (PMC10129440; doi:10.1186/s10194-023-01581-6)
Supplement: Supplementary file 2 — Additional file 2: Table S2. The number of NDPH and HCs in different neuromagnetic activity areas. HCs, healthy controls; NDPH, new daily persistent headache; All, all brain regions; L_F, left frontal lobe; R_F, right frontal lobe; L_P, left parietal lobe; R_P, right parietal lobe; L_O, left occipital lobe; R_O, right occipital lobe; L_T, left temporal lobe; R_T, right temporal lobe; *, p<0.05. [file 10194_2023_1581_MOESM2_ESM.docx]

| **Table S2 The number of NDPH and HCs in different neuromagnetic activity areas** | | | | | | | | | |
| --- | --- | --- | --- | --- | --- | --- | --- | --- | --- |
| **Group** | **L_F** | **R_F** | **L_P** | **R_P** | **L_O** | **R_O** | **L_T** | **R_T** | ***p*-value** |
| Delta (1-4Hz) |  |  |  |  |  |  |  |  | 0.703 |
| NDPH | 5 (17.86%) | 5 (17.86%) | 3 (10.71%) | 3 (10.71%) | 4 (14.29%) | 4 (14.29%) | 2 (7.14%) | 2 (7.14%) |  |
| Healthy Controls | 9 (24.32%) | 7 (18.92%) | 2 (5.41%) | 4 (10.81%) | 4 (10.81%) | 1 (2.70%) | 5 (13.51%) | 5 (13.51%) |  |
| Theta (4-8Hz) |  |  |  |  |  |  |  |  | 0.927 |
| NDPH | 8 (28.57%) | 4 (14.29%) | 2 (7.14%) | 2 (7.14%) | 3 (10.71%) | 2 (7.14%) | 2 (7.14%) | 5 (17.86%) |  |
| Healthy Controls | 9 (24.32%) | 6 (16.21%) | 2 (5.41%) | 3 (8.11%) | 4 (10.81%) | 5 (13.51%) | 5 (13.51%) | 3 (8.11%) |  |
| Alpha (8-13Hz) |  |  |  |  |  |  |  |  | 0.589 |
| NDPH | 3 (10.71%) | 4 (14.29%) | 4 (14.29%) | 2 (7.14%) | 1 (3.57%) | 1 (3.57%) | 5 (17.86%) | 8 (28.57%) |  |
| Healthy Controls | 6 (16.22%) | 8 (21.62%) | 3 (8.11%) | 3 (8.11%) | 5 (13.51%) | 3 (8.11%) | 3 (8.11%) | 6 (16.22%) |  |
| Beta (13-30Hz) |  |  |  |  |  |  |  |  | 0.624 |
| NDPH | 5 (17.86%) | 4 (14.29%) | 4 (14.29%) | 4 (14.29%) | 2 (7.14%) | 1 (3.57%) | 5 (17.86%) | 3 (10.71%) |  |
| Healthy Controls | 10 (27.03%) | 9 (24.32%) | 1 (2.70%) | 3 (8.11%) | 3 (8.11%) | 2 (5.41%) | 4 (10.81%) | 5 (13.51%) |  |
| Gamma (30-80Hz) |  |  |  |  |  |  |  |  | 0.847 |
| NDPH | 5 (17.86%) | 8 (28.57%) | 3 (10.71%) | 3 (10.71%) | 1 (3.57%) | 3 (10.71%) | 3 (10.71%) | 2 (7.14%) |  |
| Healthy Controls | 7 (18.92%) | 11 (29.73%) | 5 (13.51%) | 1 (2.70%) | 2 (5.41%) | 2 (5.41%) | 3 (8.11%) | 6 (16.22%) |  |
| Ripple (80-200Hz) |  |  |  |  |  |  |  |  | 0.027* |
| NDPH | 10 (35.71%) | 1 (3.57%) | 2 (7.14%) | 3 (10.71%) | 3 (10.71%) | 1 (3.57%) | 4 (14.29%) | 4 (14.29%) |  |
| Healthy Controls | 5 (13.51%) | 12 (32.43%) | 1 (2.70%) | 3 (8.11%) | 1 (2.70%) | 5 (13.51%) | 5 (13.51%) | 5 (13.51%) |  |
| HCs, healthy controls; NDPH, new daily persistent headache; All, all brain regions; L_F, left frontal lobe; R_F, right frontal lobe; L_P, left parietal lobe; R_P, right parietal lobe; L_O, left occipital lobe; R_O, right occipital lobe; L_T, left temporal lobe; R_T, right temporal lobe; *, *p*<0.05. | | | | | | | | | |
